# Gamma camera-specific reference standards for radioactive iodine uptake measurements

**DOI:** 10.1186/s40658-023-00575-2

**Published:** 2023-09-13

**Authors:** Jurgen E. M. Mourik, Mark Derks, Erik T. te Beek, Marc R. J. ten Broek

**Affiliations:** 1 Department of Nuclear Medicine, Franciscus Gasthuis & Vlietland Hospital, Kleiweg 500, 3045 PM Rotterdam, The Netherlands; 2 Department of Nuclear Medicine, IJsselland Hospital, Capelle aan den IJssel, The Netherlands; 3 Department of Nuclear Medicine, Reinier de Graaf Hospital, Delft, The Netherlands

**Keywords:** Radioiodine uptake test, RAIU, Gamma camera, Reference standard, Region-of-interest, Isotope formulation

## Abstract

**Background:**

Current guidelines of the radioiodine uptake (RAIU) test allow the use of different equipment, isotopes, activity and region-of-interest (ROI). We evaluated presence and extent of these differences in clinical practice and evaluated the effect of some of these variations on RAIU outcomes. Also, gamma camera-specific reference standards were calculated and retrospectively compared with measurements obtained during clinical RAIU tests.

**Materials and methods:**

First, questionnaires were sent to Dutch nuclear medicine departments requesting information about equipment usage, isotope, isotope formulation, activity and measurement techniques. Secondly, a neck phantom containing a range of activities in capsule or water-dissolved formulation was scanned. Counts were measured using automatic ROI, square box ROI or all counts in the image. Thirdly, clinical RAIU data were collected during 2015–2018 using three different gamma cameras. Reference standards for each scanner were calculated using regression analysis between reference activity and measured counts. Uptake measurements using this gamma camera-specific reference standard were compared with original measurements.

**Results:**

The survey demonstrated significant differences in isotope, isotope formulation, activity, use of neck phantoms, frequency and duration of reference measurements, distance to collimator, use of background measurements and ROI delineation. The phantom study demonstrated higher counts for the water-dissolved formulation than capsules using both automatic and square box ROI. Also, higher counts were found using a square box ROI than an automatic ROI. The retrospective study showed feasibility of RAIU calculations using camera-specific reference standards and good correlation with the original RAIU measurements.

**Conclusions:**

This study demonstrated considerable technical variation in RAIU measurement in clinical practice. The phantom study demonstrated that these differences could result in differences in count measurements, potentially resulting in different dose calculations for radioactive iodine therapy. Retrospective data suggest that camera-specific reference standards may be used instead of individual reference measurements using separate activity sources, which may thus eliminate some sources of variation.

## Background

Quantification of iodine uptake in the thyroid gland is widely used to differentiate etiology of thyrotoxicosis and to guide individual dose selection for ^131^I therapy of Graves’ disease, toxic adenoma or multinodular goiter [1, 2]. However, clinical superiority of dosimetry-guided individually calculated radioiodine dosages versus fixed dosages has not been unequivocally demonstrated [3–5]. Studies comparing different dosages are limited in number, show large clinical heterogeneity and use different methods to determine radioiodine activity [3, 6]. As a result, a wide variation in routine clinical practice of radioiodine therapy and dosimetry can be found among European treatment centers [7–10]. Although fixed doses are easier to implement, calculated doses will result in some patients receiving lower radioiodine doses than in fixed dose regimes, which adheres more to the ALARA principle [11–13]. Current guidelines allow both calculated and fixed doses [14, 15] and consider dosimetry for therapy of benign thyroid disease to be optional [16]. For either treatment strategy, radioiodine uptake measurement is recommended to ensure that the thyroid gland is capable of trapping iodine [4] and has the additional benefit of helping to determine the cause of hyperthyroidism [17].

Current guidelines for measurement of thyroidal radioiodine uptake (RAIU) recommend count measurements of the thyroid gland (*C*_thyroid_) using a thyroid probe or gamma camera usually 4–6 h and 24 h after administration of a small dose of ^123^I or ^131^I [18, 19]. For intrapatient background correction, count measurements are also taken over tissue distant from the thyroid gland (*C*_background patient_). As a reference for measurement consistency and confirmation of sensitivity, additional count measurements are taken over a second activity source containing the same radionuclide and identical amount of activity, placed inside a standardized neck phantom (*C*_phantom_) using the same geometry with background correction (*C*_background phantom_) [18]. RAIU is calculated as [14, 19]:

1 $${\text{RAIU}}\left( \% \right) = \frac{{C_{{{\text{thyroid}}}} - C_{{{\text{background}}\,\,{\text{patient}}}} }}{{C_{{{\text{phantom}}}} - C_{{{\text{background}}\,\,{\text{phantom}}}} }} \times 100$$

Recent surveys in UK [20], France [21] and Germany [22] have shown large variation in RAIU measurement techniques in routine clinical practice, including differences in equipment and software, isotope, dosages, collimators, time points, measurement duration and region-of-interest (ROI) delineation, all of which may affect measurement outcome. A potential strategy for further standardization of RAIU measurement is the elimination of the need for (and use of) reference source measurements [23, 24]. This strategy will, however, result in larger impact of equipment-related sources of error and is thus dependent on accuracy and reproducibility of gamma cameras and dose calibrators. Here, we propose to confirm the linearity of modern gamma cameras and employ this linearity to calculate gamma camera-specific reference standards. RAIU measurement using these calculated reference standards (C_*reference*_) may then be simplified as:

2 $${\text{RAIU}}\left( \% \right) = \frac{{C_{{{\text{thyroid}}}} - C_{{{\text{background}}\,\,{\text{patient}}}} }}{{C_{{{\text{reference}}}} }} \times 100$$

As previous surveys on RAIU measurement practices [20–22] did not focus specifically on the reference source measurements, we first conducted a survey among all nuclear medicine departments in Dutch hospitals to identify technical variations in performing RAIU and reference standard measurements. Secondly, we performed a phantom study to evaluate variation in measurement outcomes using different isotope formulations, different activity dosages and different region-of-interest definitions to quantify the potential for reduction in variability. Lastly, we calculated specific reference standards for three gamma cameras and compared RAIU measurements using these calculated reference standards (Eq. 2) with RAIU calculations using neck phantom activity sources (Eq. 1) obtained in a cohort of clinical RAIU tests in patients with hyperthyroidism prior to ^131^I therapy from a 3-year period.

## Methods

### Dutch survey

Questionnaires were sent to all Dutch hospitals with a nuclear medicine department, which includes 34 medium- to large-sized community hospitals and eight academic medical centers. Information was requested about equipment type (thyroid probe or gamma camera), isotope (^123^I or ^131^I), formulation of isotope (capsule or dissolved in water), amount of activity, use of a neck phantom, frequency of reference phantom measurements, distance between the neck phantom and collimator head, duration of measurement, use of background correction and specifics of region-of-interest (ROI) measurement.

### Phantom study

**Fig. 1:**
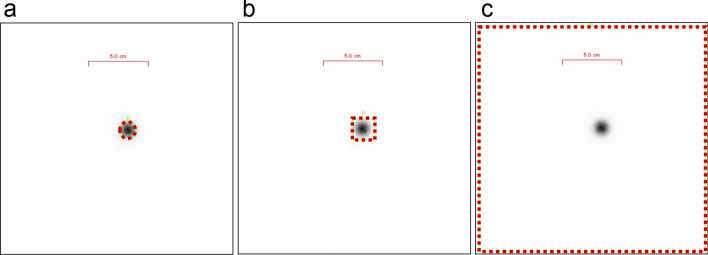
Methods for ROI delineation: automatic ROI delineation using a fixed threshold of 27% of peak activity (**a**), a square box ROI around the activity (**b**) or all counts in the image (**c**)

A neck phantom (Capintec Inc., Florham Park, USA) containing different amounts of activity was scanned with a 2011 Siemens Symbia T2 gamma camera (Siemens AG, Erlangen, Germany) using a low-energy high resolution (LEHR) collimator. Activity of ^123^I ranged from 2.3 to 15.9 MBq and was confirmed before each measurement using a calibrated well-counter (Comecer, Italy). Measurements with a fixed duration of 2 min were performed using ^123^I both in a capsule formulation and dissolved in water. Measurements of the capsule formulation were performed with a ^123^I capsule placed in a specific Perspex insert in the neck phantom. Measurements of water-dissolved ^123^I were performed after the ^123^I capsule was placed in a standard vial, water was added and then the vial was placed in the neck phantom after the capsule had completely dissolved. Distance between the neck phantom and the collimator head was fixed at 10 cm with 0 degree angle. Counts were measured using three different regions-of-interest (ROI): (1) automatic ROI delineation using a fixed threshold of 27% of peak activity, which is current clinical practice in the Franciscus Gasthuis & Vlietland Hospital, (2) a square box placed around the activity, and (3) all counts in the entire image (Fig. 1). No background correction was applied.

### Retrospective clinical data

Scan data were collected from RAIU tests performed in patients with hyperthyroidism prior to therapy with ^131^I performed between March 2015 and December 2018 at the Franciscus Gasthuis & Vlietland Hospital and IJsselland Hospital, both located in The Netherlands.

Clinical RAIU measurements at the Franciscus Gasthuis & Vlietland Hospital were performed with the Siemens ECAM (2005) gamma camera (Siemens AG, Erlangen, Germany) and a LEHR collimator. All RAIU measurements were performed using a separate activity source as reference prior to thyroid imaging. Reference activity sources containing 20.7 ± 0.5 MBq of ^123^I (range 18.4–21.7 MBq) were dissolved in water prior to imaging. Measurements were performed using a neck phantom (Capintec Inc., Florham Park, USA), a fixed duration of 600 s, a fixed angle of 0 degrees, a fixed 10 cm distance to the collimator and automatic delineation of ROI with a fixed threshold of 27% of peak activity. No background measurements were performed. Patients received activity doses of 20.7 ± 0.6 MBq (range 18.3–22.1 MBq) of ^123^I and were scanned for a fixed duration of 600 s. All activities were measured using a calibrated well-counter (Comecer, Italy) prior to administration or scintigraphic measurement. RAIU was calculated using Eq. 1 without background corrections, i.e.,

3 $${\text{RAIU}}\left( \% \right) = \frac{{C_{{{\text{thyroid}}}} }}{{C_{{{\text{phantom}}}} }} \times 100$$

Clinical RAIU measurements at the IJsselland Hospital were performed with Siemens ECAM (2006) or Siemens Symbia T2 (2011) systems (Siemens AG, Erlangen, Germany), both with a LEHR collimator. All RAIU measurements were performed using a separate activity source as reference prior to thyroid imaging. Reference activity sources were dissolved in water prior to imaging and contained 17.4 ± 1.9 MBq (range 13.3–20.9 MBq) and 17.5 ± 1.8 MBq (range 13.8–20.2 MBq) of ^123^I for the Siemens ECAM (2006) and Symbia T2 (2011) cameras respectively. Measurements were performed using a neck phantom (Capintec Inc., Florham park, USA), a fixed duration of 60 s, a fixed angle of 0 degrees and a fixed 10 cm distance to the collimator. Patients received activity doses of 16.8 ± 1.9 MBq (range 13.2–21.3 MBq) and 16.9 ± 1.9 MBq (range 13.6–19.9 MBq) of ^123^I, respectively, and were scanned for a fixed duration of 600 s. For both reference activity and patient measurements, a square box ROI was positioned around the activity. A second square box for background measurement was placed at least 5 cm away from the thyroid or activity source. All activities were measured using a calibrated well-counter (Comecer, Italy) prior to administration or scintigraphic measurement. RAIU was calculated using Eq. 1.

Reference standards for individual gamma cameras (*C*_reference_) were calculated using linear regression between activity from the reference activity source (as measured by the calibrated well-counter prior to scintigraphic measurement) and the corresponding scintigraphically measured counts from the phantom containing the activity source (*C*_phantom_) in the retrospective cohorts of clinical RAIU measurements performed on the corresponding gamma camera. The linear equation was subsequently used to calculate uptake percentages in each patient using the administered amount of ^123^I activity and the reference standard of the corresponding gamma camera and retrospectively compared with the original RAIU measurement using a separate reference activity source in a neck phantom. In accordance with Dutch law, this study was exempt from review by the local medical ethics review committee due to the retrospective nature of the study.

### Statistical analysis

In the phantom study, regression analysis and Lin’s concordance correlation coefficient (CCC) [25] were used to compare measurements using different isotope formulations (capsule formulation or dissolved in water) and different methods for ROI delineation (automatic ROI, square box or all counts in the image). Lin’s CCC ranges between − 1 and 1, where a value of 1 represents perfect agreement, a value of − 1 represents perfect disagreement, and a value of 0 represents no agreement [25]. In the retrospective clinical study, regression analysis and Lin’s CCC were used to compare outcomes of RAIU measurements using gamma camera-specific reference standard and the original RAIU measurement using a separate reference activity source in a neck phantom.

## Results

### Dutch survey

A total of 17 Dutch hospitals responded to the survey, including 15 (out of 34) community hospitals and two (out of eight) academic medical centers (Table 1). Only two hospitals used a thyroid probe and the other 15 hospitals used gamma cameras for RAIU measurements. Three hospitals used ^131^I and 14 hospitals used ^123^I as isotope. Major differences were found in the type of isotope formulation, with five hospitals using capsules (30%), seven hospitals using radioiodine dissolved in water (41%) and five hospitals using other types (29%), including syringes containing radioiodine and radioiodine dissolved in hydrochloric acid. Major differences were also found in amounts of activity used, including 3.7 MBq ^123^I in two hospitals, 10 MBq ^123^I in one hospital, 11.1 MBq ^123^I in five hospitals, 18.5 MBq ^123^I in five hospitals, 37 MBq ^123^I in one hospital, 0.5 MBq ^131^I in two hospitals and 10 MBq ^131^I in one hospital. Neck phantoms were used in eleven hospitals (65%), while the other six hospitals (35%) did not use phantoms. Reference measurements were performed before each individual patient in four hospitals (24%), daily in six hospitals (35%), weekly in six hospitals (35%) and once every 2 weeks in one hospital (6%). Distance to the collimator head was fixed at 10 cm in seven hospitals, while the other ten hospitals used varying distances including 6, 6.7, 11.5, 12, 13, 20 and 25 cm. Duration of reference measurement was 1 min in seven hospitals (41%), 2 min in two hospitals (12%), 5 min in five hospitals (29%), 10 min in one hospital (6%) and one hospital did not use a fixed duration but instead used a fixed number of measured counts. Background measurements were performed in eleven hospitals, either in a separate scan or in the same image, while the remaining six hospitals did not perform any background correction. Region-of-interest measurement was performed using automatic ROI delineation in eight hospitals (47%), while five hospitals (29%) used a square box ROI, two hospitals (12%) used all counts in the image, and the remaining two hospitals used other methods (manual, peak ^131^I with probe).

**Table 1:** Results from the survey among Dutch hospitals with a nuclear medicine department

Survey questions	Academic medical center #1	Academic medical center #2	Community hospital #1	Community hospital #2	Community hospital #3	Community hospital #4	Community hospital #5	Community hospital #6
1. Do you perform Iodine-131 therapy (e.g., for treatment of hyperthyroidism, multi-nodular goiter of toxic adenoma)?	Yes	Yes	Yes	Yes	Yes	Yes	Yes	Yes
2. Which radionuclide do you use as reference source?	Iodine-123	Iodine-131	Iodine-123	Iodine-123	Iodine-123	Iodine-123	Iodine-123	Iodine-123
3. What amount of activity (in MBq) do you use for the reference source?	11.1	0.5	11.1	11.1	18.5	37	18.5	11.1
4. Which formulation do you use for the reference source?	Capsule	Capsule	Capsule	Capsule	Capsule	Capsule	Capsule	Capsule
5. Do you use a phantom for measurement of the reference source? If so, what kind of phantom?	Neck phantom with a compartment for a capsule	Neck phantom with a compartment for a standard vial	Neck phantom with a compartment for a standard vial	No phantom	Neck phantom with a compartment for a standard vial	Neck phantom with a compartment for a capsule	Neck phantom with a compartment for a standard vial	Neck phantom with a compartment for a capsule
6. How do you prepare the reference source (before placement in the phantom)?	Direct placement of the capsule inside the phantom	Dissolvement of the capsule in (warm) water in a standard vial	Dissolvement of the capsule in (warm) water in a standard vial	Dissolvement of the capsule in (warm) water in a standard vial	Dissolvement of the capsule in (warm) water in a standard vial	Direct placement of the capsule inside the phantom	Dissolvement of the capsule in (warm) water in a standard vial	Direct placement of the capsule inside the phantom
7. How often is a new reference source prepared?	Daily	Daily	Weekly	Weekly	Prior to each patient	Daily	Weekly	Weekly
8. How often is the reference source measured?	Daily	Prior to each patient	Weekly	Weekly	Prior to each patient	Prior to each patient	Daily	Prior to each patient
9. What distance (in cm) between phantom and collimator head is used?	10	11.5	6.7	10	10	10	12	10
10. How long is the reference source measured?	1 min	2 min	100 kilocounts	5 min	1 min	5 min	10 min	1 min
11. Do you perform a background measurement?	No	Yes, in a separate background scan	No	Yes, in a separate background scan	Yes, in the neck phantom image	No	Yes, in a separate background scan	No
12. What method is used for determining the number of counts of the reference source?	Manual ROI	Total counts in the whole image	Automatic ROI	Square box ROI	Square box ROI	Automatic ROI	Automatic ROI	Automatic ROI
13. What type of equipment do you use for RAIU measurement?	Siemens Symbia T6	Siemens Symbia and Symbia Intevo	Siemens Symbia	Siemens Symbia S or Symbia T	Siemens Symbia and Symbia Intevo	Siemens Symbia T and Symbia Intevo	Siemens Symbia T2	Siemens Symbia T2

Survey questions	Community hospital #7	Community hospital #8	Community hospital #9	Community hospital #10	Community hospital #11	Community hospital #12	Community hospital #13	Community hospital #14	Community hospital #15
1. Do you perform Iodine-131 therapy (e.g., for treatment of hyperthyroidism, multi-nodular goiter of toxic adenoma)?	Yes	Yes	Yes	Yes	Yes	Yes	Yes	Yes	Yes
2. Which radionuclide do you use as reference source?	Iodine-131	Iodine-123	Iodine-123	Iodine-131	Iodine-123	Iodine-123	Iodine-123	Iodine-123	Iodine-123
3. What amount of activity (in MBq) do you use for the reference source?	10	18.5	18.5	0.5	3.7	10	11.1	3.7	18.5
4. Which formulation do you use for the reference source?	Syringe	Capsule	Capsule	Capsule	Capsule	Syringe	Capsule	Capsule	Capsule
5. Do you use a phantom for measurement of the reference source? If so, what kind of phantom?	No phantom	No phantom	Neck phantom with a compartment for a standard vial	Neck phantom with a compartment for a standard vial	Neck phantom with a compartment for a standard vial	No phantom	Neck phantom with a compartment for a capsule	No phantom	No phantom
6. How do you prepare the reference source (before placement in the phantom)?	Not applicable	Dissolvement of the capsule in (warm) water in a standard vial	Dissolvement of the capsule in (warm) water in a standard vial	Dissolvement of the capsule in hydrochloric acid	Direct placement of the capsule in a vial inside the phantom	Placement of syringe on shoulder of patient	Direct placement of the capsule inside the phantom	Not applicable	Not applicable
7. How often is a new reference source prepared?	One every two weeks	Daily	Weekly	Daily	Prior to each patient	Prior to each patient	Weekly	Prior to each patient	Daily
8. How often is the reference source measured?	Prior to each patient	Daily	Weekly	Prior to each patient	Prior to each patient	Prior to each patient	Weekly	Prior to each patient	Prior to each patient
9. What distance (in cm) between phantom and collimator head is used?	Similar distance to the patient scan procedure	10	10	25	20	As close to patient as possible	6	Not applicable	13
10. How long is the reference source measured?	1 min	5 min	1 min	5 min	5 min	2 min	1 min	Reference source is scanned together with the patient	1 min
11. Do you perform a background measurement?	Yes, in a separate background scan	No	Yes, in the neck phantom image	Yes, in the patient image	Yes, in a separate background scan	Yes, in the patient image	Yes, in the neck phantom image	Yes, in the patient image	No
12. What method is used for determining the number of counts of the reference source?	Automatic ROI	Automatic ROI	Square box ROI	Probe, Iodine-131 peak (330–418 keV)	Total counts in the whole image	Square box ROI	Automatic ROI	Square box ROI	Automatic ROI
13. What type of equipment do you use for RAIU measurement?	MED Nuklear-Medizintechnik upt2000	Siemens Symbia S	Siemens Symbia S and Symbia Intevo	Canberra Unispec	Siemens Intevo	Siemens Symbia T	GE NM/CT 870 DR	GE 640/670	Siemens Symbian Intevo

### Phantom study

**Fig. 2:**
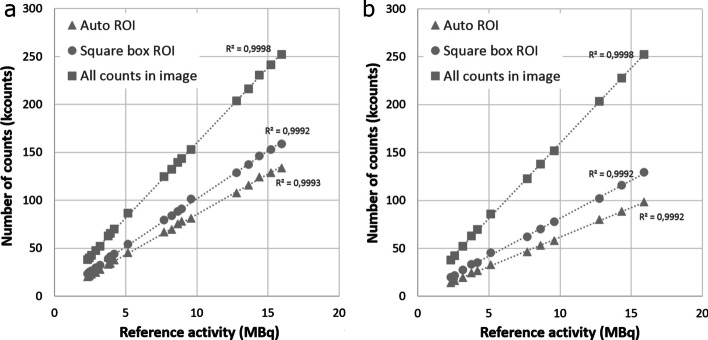
Correlation between activity and measured counts with ^123^I both in capsule formulation (**a**) and dissolved in water (**b**) using different methods of ROI delineation: automatic ROI (triangles), square box ROI (circles) and all counts in the image (squares)

**Fig. 3:**
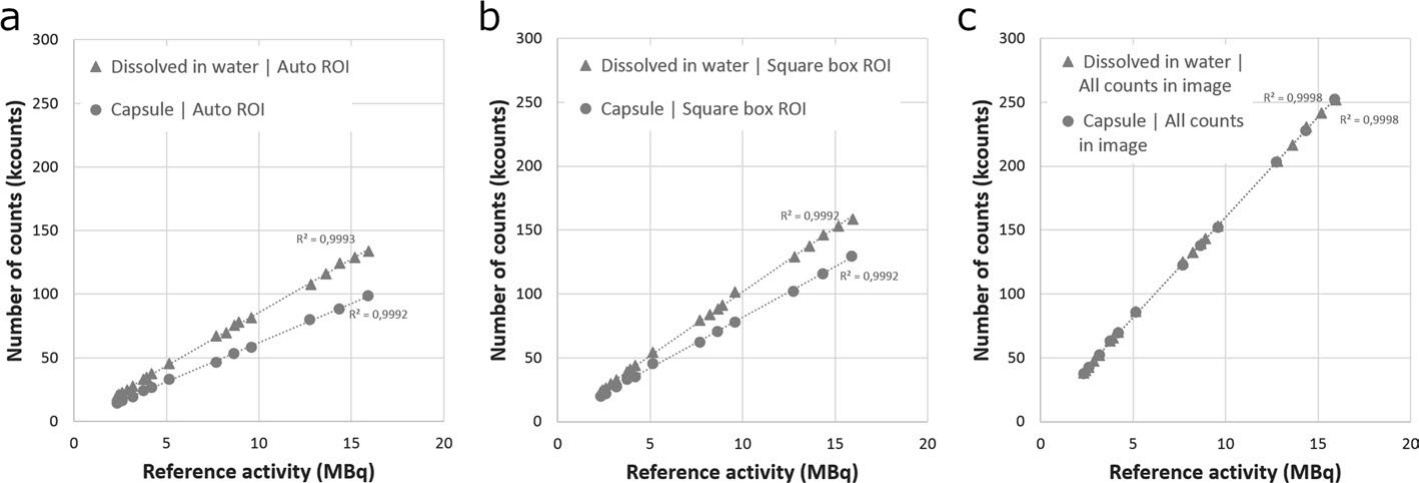
Correlation between activity and measured counts with automatic ROI delineation (**a**), square box ROI (**b**) and all counts in the image (**c**) using different isotope formulations: capsule formulation (circles) and water-dissolved formulation (triangles)

Strong linear correlations between activity and measured counts were found using both capsule formulations and water-dissolved formulations and using all three ROI delineation methods (automatic ROI, square box ROI and all counts in the image) (*R*^2^ = 1.0, Fig. 2). Compared with automatic ROI delineation, higher counts were measured using the square box ROI (26% using capsule formulation and 19% using water-dissolved formulation) and even higher counts using all counts in the image (157% using capsule formulation and 87% using water-dissolved formulation) (Fig. 2). Compared with the capsule formulation, up to 37% higher counts were measured using ^123^I dissolved in water using both automatic ROI delineation and square box ROI (Lin’s concordance correlation coefficient 0.79 and 0.88) (Fig. 3). No difference in counts measurements was found comparing capsule formulation and water-dissolved formulation using all counts in the image (Lin’s CCC 1.00).

### Retrospective study for calculation of gamma camera-specific reference standards

From 2015 to 2018, a total of 225 RAIU measurements were performed in both hospitals, consisting of 225 planar thyroid scintigrams and 225 reference source scintigrams. At the Franciscus Gasthuis & Vlietland Hospital, 116 RAIU measurements were performed on the Siemens ECAM (2005) scanner using automatic ROI delineation. At the IJsselland Hospital, 55 RAIU measurements were performed on the Siemens ECAM (2006) scanner and 54 RAIU measurements on the Siemens Symbia T2 (2011) scanner, all using square box ROI method with intrapatient and background correction.

**Fig. 4:**
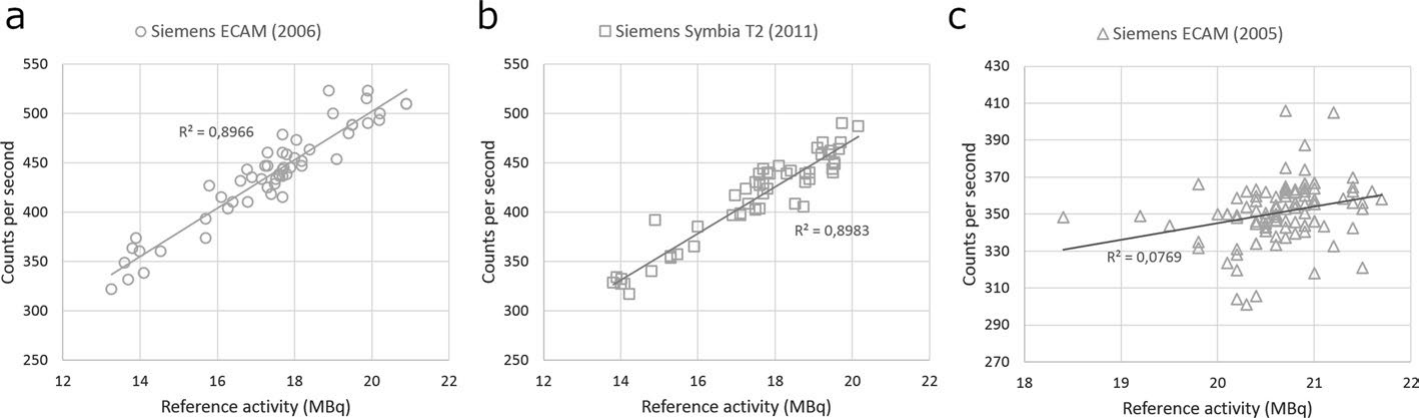
Linear relationship between activity and count rates for the Siemens ECAM (2006) (**a**), Siemens Symbia T2 (2011) (**b**) and Siemens ECAM 2005 (**c**) systems. Reference source activity from clinical RAIU measurements at the Franciscus Gasthuis & Vlietland Hospital was obtained with the Siemens ECAM (2005) gamma camera using automatic delineation of ROI without background measurements. Reference source activity from the clinical RAIU measurements at the IJsselland Hospital was obtained with both Siemens ECAM (2006) and Siemens Symbia T2 (2011) gamma cameras using a square box ROI with background measurements. All measurements were performed using a LEHR collimator

**Fig. 5:**
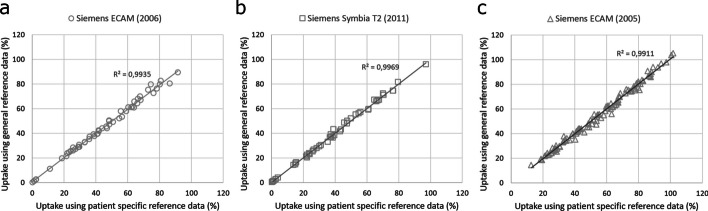
Correlation between RAIU calculations using gamma camera-specific reference standards and original RAIU calculations using individual neck phantom measurements with separate activity sources as reference per individual patient, using the Siemens ECAM (2005) (**a**), Siemens ECAM (2006) (**b**) and Siemens Symbia T2 (2011) (**c**) systems

Regression analysis of activity and measured counts of the reference source yielded linear relationships between activity and count rates for the Siemens ECAM (2005) (*R*^2^ = 0.0769), Siemens ECAM (2006) (*R*^2^ = 0.8966) and Siemens Symbia T2 (2011) systems (*R*^2^ = 0.8983) (Fig. 4). Different linear relationships were found for each gamma camera, reflecting not only the use of different gamma cameras, but also different methods for ROI delineation and use of background correction. These individual linear relationships were then used as gamma camera-specific reference for re-calculation of RAIU. Retrospective comparison of RAIU calculations using these gamma camera-specific reference standards and the original RAIU calculations using individual neck phantom measurements with separate activity sources as reference per individual patient, demonstrated Lin’s CCC of 0.9911 for RAIU measurements on the Siemens ECAM 2005, Lin’s CCC of 0.9935 for RAIU measurements on the Siemens ECAM 2006 and Lin’s CCC of 0.9969 for RAIU measurements on the Siemens Symbia T2 2011 (Fig. 5).

## Discussion

Our survey among nuclear medicine departments at Dutch hospitals, despite moderate response in a fairly small sample size, clearly showed significant differences in technical performance of RAIU test examinations in routine clinical practice, including differences in equipment, isotope, isotope formulation, amount of activity, use of neck phantoms, frequency and duration of reference measurements, distance to collimator head, background measurements and region-of-interest delineation. These results are in line with previous studies from the UK [20], France [21] and Germany [22]. The study from the UK was a national audit of thyroid uptake imaging that showed large variation in software, isotope, dosages, collimators, duration and ROI delineation and concluded that most UK centers do not adhere to all aspects of BNMS guidelines [20]. The study from France was a nationwide survey that also showed significant variation in isotope, dosages, time points of measurement post-injection and method of thyroid volumetry (ultrasound, scintigraphy or palpation) [21]. The study from Germany showed relative uniformity in equipment (majority using probe and minority using gamma camera), isotope (almost exclusively ^131^I) and amount of activity, but large variation in number and time points of measurements, distance to probe or gamma camera, software and intended organ dose [22]. These studies did not specifically focus on the reference source measurement. Our survey confirms that significant variations in clinical RAIU measurement techniques also extend into the practice and use of reference source measurements. Current guidelines [15, 18, 19] do allow the use of different equipment, isotopes, activity dosages and do not specify region-of-interest measurements, but in routine clinical practice RAIU measurements seem to be performed with even greater technical variability.

Previous studies have shown that repeated duplicate RAIU measurements are reproducible and precise [26, 27], but RAIU measurements over longer time periods may vary significantly, possibly due to activity of the autoimmune process in Graves’ disease, stress factors surrounding clinical set-up and day-to-day variations in dietary iodine intake [28]. Furthermore, the use of different equipment, such as probe systems versus gamma cameras [29], calibration of equipment [30–33], use of phantoms [34] and method of ROI delineation [35, 36] may all significantly affect measurement outcomes. Radiopharmaceuticals other than ^123^I and ^131^I have also been used for RAIU calculation, including ^124^I and ^99m^Tc-pertechnetate [37–39]. Next to all these factors, the use of RAIU and thyroid mass for subsequent calculation of therapeutic activity dose introduces several additional sources of variation. The use of a single time point (usually after 24 h) to describe iodine kinetics in the thyroid gland may be inaccurate as the biological half-life of radioiodine is variable [4]. Measurement of thyroid mass (or volume) by physical examination, scintigraphy or ultrasound examination is an important source of inaccuracy [40–42], while more recently SPECT imaging [43] or low dose CT at ^124^I-PET imaging [37] have also been proposed as suitable alternatives for thyroid volumetry. Given this multitude of sources of variation, the clinical impact of variability of individual parameters may arguably be limited, but the addition of multiple variations in performing RAIU measurements may altogether significantly limit reproducibility over time within single institutions and comparison between RAIU measurements from different hospitals. Conversely, optimal reduction of variability of as many individual parameters as possible will all contribute in enhancing accuracy and reproducibility of RAIU.

Our phantom study was performed to evaluate the extent to which technical variations, including different isotope formulations (capsule or water-dissolved formulation) and different ROI delineation methods (automatic ROI, square box ROI and all counts in the image), may affect count measurements. It is well known that the method of ROI delineation (such as manual placement, fixed dimensions or threshold based) has a direct and significant influence on quantification outcome [35, 36]. Reasons for adopting a certain ROI strategy are usually of a practical nature, such as simplicity and user-(in)dependency [36]. Using all counts in the image as ROI, with only the position of the field-of-view as variable, was included in our phantom study as reference, although current guidelines [18, 19] do not recommend this strategy for quantification, due to inaccuracy resulting from activity in neighboring tissues in the field-of-view, notably the salivary glands [44]. Compared with automatic ROI delineation, higher counts were measured using the square box ROI (up to 26% using capsule formulation and 19% using water-dissolved formulation) and, not surprisingly, even higher counts were measured using all counts in the image. Compared with the capsule formulation, higher counts were measured using the water-dissolved formulation using both automatic ROI and square box ROI (up to 37% higher) and, again not surprisingly, no difference was found using all counts in the image. These differences in count rates may be explained by the star artifact. The star artifact is caused by high energy gamma rays penetrating through the thin septa of the LEHR collimator with subsequent registration in adjacent photomultiplier tubes [45]. Capsule formulation has a higher concentration of radioiodine resulting in a more pronounced star artifact and a larger effect on count rates both within and without the ROI. With a progressively smaller ROI, progressively more counts caused by the star artifact will fall outside the ROI. Relative underestimation of count numbers of the reference standard will result in overestimation of thyroidal iodine uptake. Significant variance in count measurements and RAIU calculation will ultimately result in variance in calculation of radioiodine dose, which is inversely proportional to RAIU [14, 19]. The star artifact can be avoided by use of the ME collimator, which, however, may not be widely available and spatial resolution is generally lower [45, 46].

Our retrospective clinical dataset of 225 RAIU tests was used to evaluate the relationships between activity and count rates for three gamma camera systems. Two RAIU measurements yielded outcomes higher than 100%, probably resulting from the lack of use of intrapatient and background correction and thereby underscoring the importance of background correction. Using regression analysis, different linear relationships were found for each gamma camera, with well-fitted regression lines for the Siemens ECAM (2006) and Siemens Symbia T2 (2011) cameras (*R*^2^ = 0.8966 and *R*^2^ = 0.8983, respectively) but a lesser fitted regression line for the Siemens ECAM (2005) (*R*^2^ = 0.0769), reflecting not only the use of different gamma cameras, but also different methods for ROI delineation and use of background correction. These individual linear relationships were then used as gamma camera-specific reference standard (*C*_reference_) for re-calculation of individual RAIU with Eq. 2. Outcomes were compared with the original RAIU calculations using the individual neck phantom measurements with separate activity sources as reference, which showed excellent correlation for all three gamma cameras. Our results are in line with a previous study in 50 patients, designed to compare probe and gamma camera measurements [23], which also showed linear relationship between reference activity and measured count rate (calibration curve) with reproducibility over time and suggested that gamma camera-based RAIU measurement can be accurately performed without the use of reference activity sources. The rationale of performing individual phantom measurements as reference is confirmation of consistency and sensitivity of the measurement system during in vivo assessments [19]. For purposes of dosimetry, the number of detected counts by a gamma camera within a specified ROI should be proportional to the activity within that ROI, but at high activity levels the system dead time can lead to substantial count rate losses [47, 48] with resultant nonlinearity. Our results and those of others [23] suggest that current gamma camera systems have only limited variation in the linear relationship between activity and measured count rates, within the activity range relevant for RAIU testing. Furthermore, calibration factors and gamma camera sensitivity have been shown to be stable over time [49]. Thus, given the stability of linear calibration and reproducibility over time, RAIU calculation with gamma camera-specific reference standards is feasible. Elimination of individual phantom reference measurements may reduce some of the technical variations in routine clinical practice. In addition, reduction in phantom reference measurements may also result in lower costs, gamma camera time occupancy and radiation exposure of personnel. Periodic, highly standardized quality control at low frequency may then suffice [31, 50, 51]. An additional benefit may be that equal calibration of different gamma camera systems with similar linear regression lines for activity and count rate relationships may allow the interchangeable use of different gamma cameras for clinical RAIU measurements within single institutions or even between different hospitals [52]. However, a reduction in the use of individual reference phantom measurements will inevitably result in larger impact of radioiodine under- or over-dosing due to variability in measurement accuracy of radionuclide calibrators, which can be substantial [33]. Therefore, the use of well-calibrated and stable radionuclide calibrators as well as standardized sample configuration and detector geometry are essential prerequisites.

A limitation of this study is that only ^123^I was evaluated. Many institutions use ^131^I for reasons of availability, costs and the longer half-life. Also, the evaluation of the effect of different types of ROI delineation on count rate measurement was limited to square box ROI delineation and automatic ROI delineation with a fixed threshold of 27% of peak activity, and did not investigate automatic ROI with lower thresholds. In addition, the geometry of square box ROI delineation is not standardized, but operator-dependent, which may have added variability.

We propose to further standardize RAIU measurements and use the linearity of modern gamma cameras to calculate gamma camera-specific reference standards. The linearity of the gamma camera should be confirmed by measurement of multiple doses (e.g., 1, 2, 4, 6 and 10 MBq of ^123^I) using an activity source in water-dissolved formulation inside a standard neck phantom at 10 cm distance to the collimator head with a fixed measurement duration of 1 min. ROI delineation should also be standardized. As geometry of the square boxes and thresholds of automatic ROI delineations may vary, our current data do not suggest any preference of one method over another, but consistent use of one sharply defined method is essential. The use of intrapatient background correction is emphasized to avoid the star artifact from the thyroid uptake.

## Conclusion

Our study shows significant variability in technical aspects of performing RAIU measurements in routine clinical practice and confirms for several of these variations their effects on count rates. For three gamma camera systems, we have confirmed a specific linear relationship between activity and measured count rate, which enables calculation of gamma camera-specific reference standards. We have demonstrated that RAIU calculation with gamma camera-specific reference standards is feasible and shows excellent correlation with the original RAIU measurement outcomes.

We propose to further standardize RAIU measurements and use the linearity of modern gamma cameras to calculate gamma camera-specific reference standards, which may obviate the need for separate (phantom) reference source measurements. The linearity of the gamma camera should be confirmed and periodic, highly standardized quality control at low frequency may then suffice. The use of well-calibrated and stable radionuclide calibrators with standardized sample configuration and detector geometry, are essential prerequisites. For further standardization, optimal methods for ROI delineation and thyroid volumetry need to be defined, also with regard to the improved contrast and spatial resolution of new generation gamma cameras [53]. Lastly, the role of quantitative SPECT [54] and deep-learning technology [55] for RAIU measurement and their potential for reduction of measurement variability need to be further evaluated in large patient cohorts.

## Data Availability

The datasets used and/or analyzed during the current study are available from the corresponding author on reasonable request.
